# Cerebral endothelial dysfunction in reversible cerebral vasoconstriction syndrome: a case-control study

**DOI:** 10.1186/s10194-017-0738-x

**Published:** 2017-02-23

**Authors:** Hyun Ah Choi, Mi Ji Lee, Chin-Sang Chung

**Affiliations:** Samsung Medical Center, Sungkyunkwan University School of Medicine, Neurology, 81 Irwon-Ro, Gangnam-Gu, Seoul, 06531 South Korea

**Keywords:** Reversible cerebral vasoconstriction syndrome, Pathophysiology, Endothelial dysfunction, Breath holding index, Cerebral vasomotor reactivity

## Abstract

**Background:**

The aim of this study is to investigate cerebral endothelial dysfunction in patients with reversible cerebral vasoconstriction syndrome (RCVS).

**Methods:**

We prospectively recruited patients with RCVS, age-matched controls with episodic migraine, and age-matched healthy controls at Samsung Medical Center from Apr 2015 to Jul 2016. All participants underwent transcranial Doppler evaluation, with a breath-holding maneuver, for the evaluation of bilateral middle cerebral arteries (MCAs), posterior cerebral arteries (PCAs), and the basilar artery (BA). The breath-holding index (BHI) was used to measure cerebral endothelium-dependent vasodilation. Follow-up BHIs were recorded in selected patients with RCVS after 3 months.

**Results:**

A total of 84 subjects were recruited for this study (*n* = 28 in each group of RCVS, episodic migraine, and healthy control; mean age, 49.8 years). The RCVS group showed lower BHIs in all basal arteries, in comparison to healthy controls (*p* < 0.001, 0.009 for bilateral MCAs, *p* < 0.001 and 0.028 for bilateral PCAs, and *p* = 0.060 for the BA). Compared to migraineurs, RCVS patients had lower BHIs only in the anterior circulation (*p* = 0.002 and 0.038 for bilateral MCAs; *p =* 0.069 and 0.247 for bilateral PCAs; *p =* 0.120 for the BA). Of the 10 patients who had follow-up BHIs at 3 months, 7 showed complete normalization, while three did not.

**Conclusions:**

Cerebral endothelial function is impaired in a widespread distribution in RCVS. Its role in the pathogenesis and clinical outcome of RCVS should be determined in further studies.

**Electronic supplementary material:**

The online version of this article (doi:10.1186/s10194-017-0738-x) contains supplementary material, which is available to authorized users.

## Background

Reversible cerebral vasoconstriction syndrome (RCVS) is characterized by reversible multifocal narrowing of the cerebral arteries with typical manifestations of recurrent thunderclap headache with or without focal neurologic deficits [1]. In patients with RCVS, thunderclap headache is often triggered by the Valsalva maneuver or emotional stress, implicating a dysregulation of cerebral arterial tone with increased sympathetic stimuli.

The mechanism of RCVS is not fully understood. To date, abnormal heart rate variability [2], reduced circulating endothelial progenitor cells [3], increased oxidative stress [4], and brain-derived neurotrophic factor (BDNF) polymorphism [5] have been reported to play a pathogenic role in RCVS. Recently, reduced cerebral vasomotor reactivity, which represents cerebral endothelial dysfunction, was found in seven of eight selected patients with RCVS in a small retrospective study [6]. However, cerebral endothelial dysfunction is also present in migraineurs, especially in the posterior circulation but also in the anterior circulation in chronic patients [7, 8]. To identify a disease-specific pattern of cerebral endothelial dysfunction, we aimed to investigate cerebral endothelial function in patients with RCVS compared with age-matched migraineurs and healthy controls.

## Methods

### Participants

We prospectively recruited patients with RCVS within 1 month of onset at the Samsung Medical Center from April 2015 to July 2016. For the control group, we recruited age-matched patients with episodic migraine without aura (MOA) and healthy controls, with a 1:1:1 patient-to-control ratio from April 2015 to July 2016. This study was approved by the Institutional Review Board in the Samsung Medical Center. All the diagnoses of RCVS and MOA were based on the third edition of the International Classification of Headache Disorders (ICHD-3 beta). Healthy controls did not have any history of migraine, hypertension, diabetes mellitus, hyperlipidemia, stroke, cardiac disease, smoking, or the use of angiotensin II receptor blocker (ARB), angiotensin converting enzyme inhibitor (ACEi) or statin treatments.

### Clinical evaluation

All patients and controls were interviewed by investigators (HAC, MJL, and C-SC), using a structured headache questionnaire on the headache characteristics, medical history, and current medications. For the RCVS group, neurological complications including focal neurological deficits, seizure, ischemic stroke, subarachnoid hemorrhage (SAH), and posterior reversible encephalopathy syndrome (PRES) were evaluated through clinical observation and neuroimaging. All patients with RCVS underwent contrast-enhanced brain magnetic resonance imaging (MRI) and magnetic resonance angiogram (MRA). Reversibility was confirmed in all patients with RCVS with a follow-up MRA 3–6 months after symptom onset.

### Measurement of cerebral endothelial dysfunction

To determine cerebral endothelial dysfunction, endothelium-dependent vasodilatory function was measured using transcranial Doppler (TCD) during a breath-holding maneuver. TCD tests were performed within 1 week of diagnosis in the RCVS group, between thunderclap headache attacks. For the MOA group, TCD assessment was performed in the interictal period following 48 h without headache. The TCD evaluation was performed using a Pioneer TC 8080 (Nicolet Vascular, Madison, WI, USA) by experienced neurophysiologists who were blinded to clinical information.

The 2-MHz Doppler hand-held probe was fixed manually in a supine position. The baseline mean flow velocity (MFV) of bilateral middle cerebral arteries (MCAs), posterior cerebral arteries (PCAs), and the basilar artery (BA) were determined initially. Patients were asked to hold their breath for 30 s and keep breathing normally until the initiation of breath-holding procedure to avoid the Valsalva manoeuver or hyperventilation. Patients who were unable to hold their breath for the required period were reassessed up to three times. MFV was thoroughly monitored and recorded at the end of the 30-s breath-holding maneuver [9–11]. The breath-holding index (BHI) was calculated as the % increment of the MFV (*i.e.*, [MFV at the end of breath-holding – baseline MFV] x 100/baseline MFV) divided by the duration of the breath-holding (30 s) [10]. The BHI was determined in bilateral MCAs, PCAs, and the BA. In the RCVS group, the BHI measurement was repeated in selected patients after 3 months.

### Statistical analyses

Statistical analyses were performed using commercially available SPSS version 18.0 software (SPSS Inc., Chicago, IL, USA). Variables were presented as a proportion, mean ± standard deviation (SD), or a median and interquartile range (IQR). Bivariate analyses were performed using Student’s t-tests and Mann-Whitney tests for continuous variables, and Chi-squared and Fisher’s exact tests for categorical variables. Independent t-tests were used for the case-control comparison of the MFVs and BHIs in each vessel. To adjust the baseline flow velocities, analysis of covariance (ANCOVA) tests were performed to compare the BHIs in each vessel. The mean BHIs in all tested arteries in each patient was used to group patients into the higher vs lower mean BHI subgroups, using the median value as a cutoff. Demographics, clinical characteristics, and neurologic complications were compared between the two groups via linear regression analysis. Two-tailed *p*-values <0.05 were used to establish a statistical significance.

## Results

### Baseline demographics of RCVS patients

A total of 34 patients with RCVS were recruited during the study period. Among them, six patients were excluded, since they had received treatment with nimodipine (*n* = 4) or refused to participate in the study (*n* = 2). Thus, 28 patients who completed the TCD assessment per protocol were included. Age-matched migraineurs and healthy controls (mean age, 49.8 years; age range, 30–63 years) were also recruited. The baseline demographics of participants are presented in Table 1. Patients with RCVS had a higher frequency of hyperlipidemia (*p =* 0.004, compared with healthy control), and thus more of these patients were currently being treated with statin medication (*p =* 0.023; Table 1) at the time of study.

**Table 1:** Baseline demographics of patients with reversible cerebral vasoconstriction syndrome, migraineurs, and healthy controls

	RCVS (*N* = 28)	Migraine (*N* = 28)	Healthy controls (*N* = 28)	*p*-value	
				vs migraine^a^	vs HC^a^
Age	49.8 ± 10.08 (30–63)	49.8 ± 10.08 (30–63)	49.8 ± 10.08 (30–63)	>0.999	>0.999
Female sex	24 (85.7%)	24 (85.7%)	25 (89.3%)	>0.999	>0.999
Diabetes mellitus	4 (14.3%)	0 (0%)	0 (0%)	0.111	0.111
Hypertension	4 (14.3%)	1 (3.6%)	0 (0%)	0.611	0.236
Hyperlipidemia	8 (28.6%)	3 (10.7%)	0 (0%)	0.177	**0.004**
Stroke	0 (0%)	0 (0%)	0 (0%)		
Cardiac disease	1 (3.6%)	0 (0%)	0 (0%)	>0.999	>0.999
Smoking	1 (3.6%)	0 (0%)	0 (0%)	>0.999	>0.999
Current medication					
ARB	1 (3.6%)	0 (0%)	0 (0%)	>0.999	>0.999
ACE inhibitor	1 (3.6%)	0 (0%)	0 (0%)	>0.999	>0.999
Statin	6 (21.4%)	1 (3.6%)	0 (0%)	0.101	**0.023**
History of migraine	6 (21.4%)	28 (100.0%)	0 (0%)	**<0.001**	**0.023**
Causes of RCVS					
Idiopathic	24 (85.7%)				
Postpartum	4 (14.3%)				
Medication	0 (0%)				
Headache					
Thunderclap onset	24 (85.7%)				
Triggered by typical precipitants	17 (60.7%)				
Recurrent during first 1 month	19 (67.9%)				
Neurological complication					
Neurological deficits	1 (3.6%)				
Seizure	3 (10.7%)				
Cerebral infarction	0 (0%)				
Cortical SAH	1 (3.6%)				
PRES	2 (7.1%)				

*ACE* angiotensin converting enzyme, *ARB* angiotensin II receptor blocker, *PRES* posterior reversible encephalopathy syndrome, *RCVS* reversible cerebral vasoconstriction syndrome, *SAH* subarachnoid hemorrhage

Values are presented as number (%), mean ± standard deviation (ranges). ^a^compared with RCVS

### Transcranial doppler findings in RCVS

Patients with RCVS underwent TCD tests at a median of 7.5 (IQR, 4.0–16.5) days after onset. There were no significant complications during the breath-holding maneuver. Patients and healthy controls tolerated the breath hold well, except one patient who complained of headache. Baseline mean flow velocities were higher in the RCVS group in bilateral MCAs and the BA, compared with migraineurs (*p =* 0.059 for the left MCA, *p =* 0.046 for the right MCA, and *p =* 0.016 for the BA; Table 2) and healthy controls (*p =* 0.032 for the left MCA, *p =* 0.041 for the right MCA, and *p =* 0.034 for the BA; Table 2). Nine of twenty-eight patients with RCVS (32.1%) presented increased MFVs in the MCAs that exceeded the upper limit of the normal range (80 cm/s). Among them, four patients (14.3%) met the criteria for mild vasospasm (>120 cm/s). There were no significant differences in the flow velocities of bilateral PCAs.

**Table 2:** Cerebral blood flow velocities and breath-holding indices

	RCVS (*N* = 28)	Migraine (*N* = 28)	Healthy controls (*N* = 28)	*p*-value (adjusted p)	
				vs migraine^a^	vs HC^a^
Mean flow velocities (cm/s)					
L MCA	76.4 ± 28.56	64.2 ± 11.20	62.5 ± 10.47	0.059	**0.032**
R MCA	78.4 ± 28.05	65.9 ± 14.04	66.1 ± 10.32	0.046	**0.041**
L PCA	38.6 ± 10.75	36.5 ± 6.65	37.4 ± 6.58	0.410	0.617
R PCA	39.4 ± 11.29	36.8 ± 6.36	38.8 ± 7.14	0.341	0.906
BA	54 ± 13.61	46.7 ± 5.98	46.5 ± 10.65	**0.016**	**0.034**
Breath-holding index					
L MCA	0.8 ± 0.33	1.1 ± 0.34	1.2 ± 0.24	**0.002** **(0.004)**	**<0.001** **(<0.001)**
R MCA	1.0 ± 0.36	1.3 ± 0.52	1.3 ± 0.39	**0.038** (0.109)	**0.009** **(0.033)**
L PCA	0.8 ± 0.42	1.0 ± 0.38	1.3 ± 0.43	0.069 (0.160)	**<0.001** **(<0.001)**
R PCA	1.0 ± 0.42	1.1 ± 0.34	1.3 ± 0.49	0.247 (0.375)	**0.028** (0.090)
BA	0.9 ± 0.47	1.0 ± 0.42	1.1 ± 0.31	0.120 (0.178)	0.060 **(0.031)**

*BA* basilar artery, HC, healthy control, *MCA* middle cerebral artery, *PCA* posterior cerebral artery, *RCVS* reversible cerebral vasoconstriction syndrome

Values are presented as mean ± standard deviation. ^a^compared with RCVS

Adjustment of flow velocities were performed to compare breath holding indices in each vessel

All patients of RCVS showed significantly lower BHIs in basal arteries compared with healthy controls. In addition, patients with RCVS showed lower BHIs in the anterior circulation compared to subjects with episodic MOA (Table 2). Differences in the BHIs between the RCVS and MOA groups were not significant in the posterior circulation, and the results were generally unchanged after controlling for baseline MFVs. Similar results were observed even when the six migraineurs in the RCVS group were excluded (Additional file 1: Table S1-S2).

### Factors related to cerebral endothelial dysfunction in RCVS

When patients with RCVS were stratified according to a higher or lower BHIs, vascular risk factors were not associated with endothelial dysfunction (Table 3). Patients with lower BHIs were younger and had more migraines than those with higher BHIs, although statistical significance between groups was not reached.

**Table 3:** Factors related to cerebral endothelial dysfunction in RCVS

	Low BHI (N = 14)	High BHI (N = 14)	*P*-value
Age	46.9 ± 10.93 (30–60)	52.7 ± 8.55 (35–63)	0.126
Female sex	13 (92.9%)	11 (78.6%)	0.297
Diabetes mellitus	2 (14.3%)	2 (14.3%)	>0.999
Hypertension	1 (7.1%)	2 (14.3%)	0.558
Hyperlipidemia	5 (35.7%)	3 (21.4%)	0.422
Stroke	0 (0%)	0 (0%)	
Cardiac disease	1 (7.1%)	0 (0%)	0.327
Smoking	0 (0%)	1 (7.1%)	0.327
Current medication			
ARB	0 (0%)	1 (7.1%)	0.276
ACE inhibitor	1 (7.1%)	0 (0%)	0.385
Statin	2 (14.3%)	4 (28.6%)	0.376
History of migraine	5 (35.7%)	1 (7.1%)	0.069
Causes of RCVS			0.297
Idiopathic	11 (78.6%)	13 (92.9%)	
Postpartum	3 (21.4%)	1 (7.1%)	
Medication	0 (0%)	0 (0%)	

*ACE* angiotensin converting enzyme, *ARB* angiotensin II receptor blocker, *RCVS* reversible cerebral vasoconstriction syndrome

Values are presented as number (%), mean ± standard deviation (ranges)

### Follow-up evaluation in RCVS

**Fig. 1:**
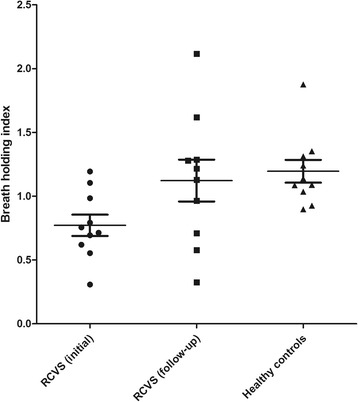
Baseline and follow-up breath holding indices in patients and matched controls Breath holding index (BHI) of all tested vessels was averaged. The follow-up study showed a complete normalization in seven patients, no change in two, and aggravation in one patient

Ten RCVS patients underwent follow-up BHI studies after 3 months. Follow-up study results showed complete normalization in seven patients (pre/post, 0.8 ± 0.22/1.3 ± 0.46), while three patients did not show a significant improvement (no change, *n* = 2; aggravation, *n* = 1) (Fig. 1).

## Discussion

The key findings of this article are as follows: (1) cerebral endothelial dysfunction is present in patients with RCVS, (2) the pattern of endothelial dysfunction is more widespread in RCVS than in migraine, and (3) endothelial dysfunction is reversible in most, but not all patients. Impaired cerebral endothelial dysfunction may play a role in the pathophysiology of RCVS; its role in predisposition should be determined in future researches.

The endothelium, which comprises the innermost layer of the vessel wall, is a key mediator of vascular tone since it controls vasoconstriction and vasodilation [12]. Endothelial cell function can be assessed in two manners. One method of assessment is measuring circulating markers of endothelial cell activation, injury, or repair [12, 13]. In a recent study, Chen et al. [3] reported a reduced number of circulating markers that is responsible for endothelial cell repair in patients with RCVS. The other method of measuring endothelial cell function is the assessment of endothelium-dependent vasomotion [12]. Unlike circulating markers, endothelium-dependent vasomotion can be measured differently in systemic and cerebral circulation. Cerebral vasomotor reactivity is a method that specifically measures endothelium-dependent vasodilation in cerebral circulation, which may differ from systemic endothelial function [14, 15]. Our findings confirm that endothelium-dependent vasodilation is impaired in the cerebral arteries of patients with RCVS. Along with the findings of cardiac involvement in RCVS [15], future research should focus on the correlation or difference between cerebral and systemic endothelial dysfunction in RCVS.

Breath holding is a physiologic and reliable method to induce endothelium-dependent vasodilation in cerebral arteries in response to arterial carbon dioxide levels [10, 16]. The BHI is calculated using the % increment of post-breath-holding MFVs compared to baseline MFVs. In the present study, patients with RCVS showed significantly reduced BHIs in all basal arteries, implying clinical significance of impaired endothelium-dependent vasodilation in RCVS. There was, however, not a great difference in the posterior circulation in RCVS compared with episodic MOA. Cerebral endothelial dysfunction in the posterior circulation has been consistently reported in migraine [8]. Two hypotheses can explain the differences in the distribution of cerebral endothelial dysfunction between RCVS and migraine. First, endothelial function may be systemically impaired in patients with RCVS to a greater extent than that in migraineurs [3, 14, 15]. Second, as the posterior circulation is less innervated with sympathetic fibers than the anterior circulation [17], both sympathetic and parasympathetic dysfunction may play a role in the pathophysiology of RCVS [2].

Our study showed that endothelial dysfunction in RCVS is not related to advanced age, vascular risk factors, or premorbid medications. Rather, younger age and premorbid migraine were associated with more severe endothelial dysfunction, although statististically insignificant. However, it is still not clear if cerebral endothelial dysfunction is a predisposition of RCVS or a part of its pathophysiology. Although our study was not longitudinally designed, our limited data showed a normalization of endothelial dysfunction in some patients. However, endothelial dysfunction was persistent or even aggravated in a small number of patients, suggesting either poor treatment outcome or premorbid endothelial dysfunction. Future studies should be designed to determine whether endothelial dysfunction is a predisposition for RCVS or not. In addition, other predisposing factors such as genetic polymorphism should be considered in association with endothelial dysfunction [5].

This study has strengths. First, this is a prospective case-control study that included RCVS, migraine, and healthy control subjects. Confounding factors that might affect cerebral microvasculature were thoroughly investigated and controlled for in the analysis. Second, we selectively measured cerebral-specific endothelial function in RCVS. Third, we made several hypotheses regarding the pathogenesis of RCVS. However, this study has some limitations. First, this study was performed in a single ethnic population, *i.e.* Asians. Our study subjects had a small number of neurological complications, which might be attributed to the study population. External validation is warranted in different populations. Second, we did not evaluate concurrent systemic endothelial dysfunction in comparison to the BHI. It would be worthwhile to test the correlation or difference between systemic endothelial dysfunction (*i.e.* flow-mediated dilatation) and the BHI in future studies.

## Conclusions

In conclusion, our results suggest that cerebral endothelial function is impaired in patients with RCVS in a widespread pattern. Future investigations should be conducted to reveal differences in systemic and cerebral endothelial function and the implications of endothelial dysfunction in the pathogenesis and neurological outcomes of patients with RCVS.

## Additional file

Additional file 1: Table S1. Baseline demographics of patients with reversible cerebral vasoconstriction syndrome, migraineurs, and healthy controls. **Table S2**. Cerebral blood flow velocities and breath-holding indices. (DOC 66 kb)

## Abbreviations

ACEi: Angiotensin converting enzyme inhibitor
ARB: Angiotensin II receptor blocker
BA: Basilar artery
BDNF: Brain-derived neurotrophic factor
BHI: Breath-holding index
ICHD-3 beta: International Classification of Headache Disorders
IQR: Interquartile ranges
MCAs: Middle cerebral arteries
MFV: Mean flow velocity
MOA: Migraine without aura
MRA: Magnetic resonance angiogram
MRI: Magnetic resonance imaging
PCAs: Posterior cerebral arteries
PRES: Posterior reversible encephalopathy syndrome
RCVS: Reversible cerebral vasoconstriction syndrome
SAH: Subarachnoid hemorrhage
SD: Standard deviation
TCD: Transcranial doppler
