# Supplementary material for: Cerebral endothelial dysfunction in reversible cerebral vasoconstriction syndrome: a case-control study
Source: J Headache Pain. 2017 Feb 23;18(1):29. doi: 10.1186/s10194-017-0738-x (PMC5321640; doi:10.1186/s10194-017-0738-x)
Supplement: Additional file 1: Table S1. — Baseline demographics of patients with reversible cerebral vasoconstriction syndrome, migraineurs, and healthy controls. Table S2. Cerebral blood flow velocities and breath-holding indices. (DOC 66 kb) [file 10194_2017_738_MOESM1_ESM.doc]

Additional file 1: Table S1. Baseline demographics of patients with reversible cerebral vasoconstriction syndrome, migraineurs, and healthy controls.

|  | Non-migraineur-RCVS (N=22) | Migraine (N=22) | Healthy controls (N=22) | p-value | |
| --- | --- | --- | --- | --- | --- |
|  |  |  |  | vs migraine* | vs HC* |
| Age | 50.2 ± 10.55 (30–63) | 50.2 ± 10.55 (30–63) | 50.2 ± 10.55 (30–63) | >0.999 | >0.999 |
| Female sex | 3 (13.6%) | 3 (13.6%) | 4 (18.2%) | >0.999 | >0.999 |
| Diabetes mellitus | 3 (13.6%) | 0 (0%) | 0 (0%) | 0.233 | 0.233 |
| Hypertension | 3 (13.6%) | 1 (4.5%) | 0 (0%) | 0.607 | 0.233 |
| Hyperlipidemia | 6 (27.3%) | 3 (13.6%) | 0 (0%) | 0.457 | **0.011** |
| Stroke | 0 (0%) | 0 (0%) | 0 (0%) |  |  |
| Cardiac disease | 1 (4.5%) | 0 (0%) | 0 (0%) | >0.999 | >0.999 |
| Smoking | 1 (4.5%) | 0 (0%) | 0 (0%) | >0.999 | >0.999 |
| Current medication |  |  |  |  |  |
| ARB | 1 (4.5%) | 0 (0%) | 0 (0%) | >0.999 | >0.999 |
| ACE inhibitor | 1 (4.5%) | 0 (0%) | 0 (0%) | >0.999 | >0.999 |
| Statin | 5 (22.7%) | 1 (4.5%) | 0 (0%) | 0.185 | **0.048** |
| Causes of RCVS |  |  |  |  |  |
| Idiopathic | 24 (85.7%) |  |  |  |  |
| Postpartum | 4 (14.3%) |  |  |  |  |
| Medication | 0 (0%) |  |  |  |  |
| Headache |  |  |  |  |  |
| Thunderclap onset | 20 (90.9%) |  |  |  |  |
| Triggered by typical precipitants | 12 (54.5%) |  |  |  |  |
| Recurrent during first 1 month | 16 (72.7%) |  |  |  |  |
| Neurological complication |  |  |  |  |  |
| Neurological deficits | 1 (4.5%) |  |  |  |  |
| Seizure | 1 (4.5%) |  |  |  |  |
| Cerebral infarction | 0 (0%) |  |  |  |  |
| Cortical SAH | 1 (4.5%) |  |  |  |  |
| PRES | 2 (9.1%) |  |  |  |  |

Values are presented as number (%), mean ± standard deviation (ranges). * compared with RCVS.

Abbreviations: ACE, angiotensin converting enzyme; ARB, angiotensin II receptor blocker; PRES, posterior reversible encephalopathy syndrome; RCVS, reversible cerebral vasoconstriction syndrome; SAH, subarachnoid hemorrhage

Table S2. Cerebral blood flow velocities and breath-holding indices.

|  | Non-migraineur-RCVS (N=22) | Migraine (N=22) | Healthy controls (N=22) | p-value | |
| --- | --- | --- | --- | --- | --- |
|  |  |  |  | vs migraine* | vs HC* |
| Mean flow velocities (cm/s) |  |  |  |  |  |
| L MCA | 75.0 ± 22.40 | 63.2 ± 10.45 | 63.2 ± 9.73 | **0.038** | **0.049** |
| R MCA | 75.1 ± 24.12 | 64.3 ± 11.74 | 66.5 ± 9.81 | 0.070 | 0.135 |
| L PCA | 36.6 ± 9.04 | 35.9 ± 5.97 | 37.2 ± 6.04 | 0.769 | 0.823 |
| R PCA | 36.7 ± 8.01 | 35.6 ± 6.25 | 39.1 ± 6.66 | 0.605 | 0.296 |
| BA | 52.0 ± 13.75 | 46.1 ± 5.67 | 45.4 ± 10.55 | 0.075 | 0.084 |
| Breath-holding index |  |  |  |  |  |
| L MCA | 0.9 ± 0.31 | 1.1 ± 0.36 | 1.2 ± 0.26 | **0.039**  (0.053) | **<0.001**  **(<0.001)** |
| R MCA | 1.1 ± 0.31 | 1.2 ± 0.48 | 1.4 ± 0.38 | 0.324  (0.389) | **0.008**  **(0.012)** |
| L PCA | 0.9 ± 0.44 | 1.0 ± 0.38 | 1.3 ± 0.43 | 0.191  (0.204) | **0.005**  **(0.005)** |
| R PCA | 1.1 ± 0.43 | 1.2 ± 0.34 | 1.3 ± 0.52 | 0.472  (0.453) | 0.068  (0.079) |
| BA | 0.9 ± 0.39 | 1.0 ± 0.40 | 1.2 ± 0.28 | 0.452  (0.513) | **0.024**  (0.088) |

Values are presented as mean ± standard deviation. * compared with RCVS.

Adjustment of flow velocities were performed to compare breath-holding indices in each vessel.

Abbreviations: BA, basilar artery; HC, healthy control; MCA, middle cerebral artery; PCA, posterior cerebral artery; RCVS, reversible cerebral vasoconstriction syndrome
